# Drought-induced recruitment of specific root-associated bacteria enhances adaptation of alfalfa to drought stress

**DOI:** 10.3389/fmicb.2023.1114400

**Published:** 2023-02-23

**Authors:** Wenqiang Fan, Fang Tang, Jiani Wang, Jiaqi Dong, Jing Xing, Fengling Shi

**Affiliations:** Key Laboratory of Grassland Resources of the Ministry of Education and Key Laboratory of Forage Cultivation, Processing and High-Efficiency Utilization of the Ministry of Agriculture, College of Grassland, Resources and Environment, Inner Mongolia Agricultural University, Hohhot, China

**Keywords:** drought stress, alfalfa (*Medicago sativa* L.), 16S rRNA sequencing, rhizosphere bacteria, plant-microbe interactions

## Abstract

Drought is a major abiotic stress that threatens crop production. Soil microbiomes are thought to play a role in enhancing plant adaptation to various stresses. However, it remains unclear whether soil microbiomes play a key role when plants are challenged by drought and whether different varieties are enriched with specific bacteria at the rhizosphere. In this study, we measured changes in growth phenotypes, physiological and biochemical characteristics of drought-tolerant alfalfa (AH) and drought-sensitive (QS) under sterilized and unsterilized soil conditions with adequate watering and with drought stress, and analyzed the rhizosphere bacterial community composition and changes using 16S rRNA high-throughput sequencing. We observed that the unsterilized treatment significantly improved the growth, and physiological and biochemical characteristics of alfalfa seedlings under drought stress compared to the sterilized treatment. Under drought stress, the fresh and dry weight of seedlings increased by 35.24, 29.04, and 11.64%, 2.74% for unsterilized AH and QS, respectively, compared to sterilized treatments. The improvement was greater for AH than for QS. AH and QS recruited different rhizosphere bacteria when challenged by drought. Interestingly, under well-watered conditions, the AH rhizosphere was already rich in drought-tolerant bacterial communities, mainly *Proteobacteria* and *Bacteroidetes*, whereas these bacteria started to increase only when QS was subjected to drought. When drought stress was applied, AH was enriched with more drought-tolerant bacteria, mainly *Acidobacteria*, while the enrichment was weaker in QS rhizosphere. Therefore, the increase in drought tolerance of the drought-tolerant variety AH was greater than that of the drought-sensitive variety QS. Overall, this study confirmed the key role of drought-induced rhizosphere bacteria in improving the adaptation of alfalfa to drought stress, and clarified that this process is significantly related to the variety (genotype). The results of this study provide a basis for improving drought tolerance in alfalfa by regulating the rhizosphere microbiome.

## Introduction

Global climate change exacerbates the frequency and duration of drought stress, which severely impacts crop yield and quality. Drought can reduce yields of major crops by 50–80% (Griffiths and Paul, 2017; Lamaoui et al., 2018). Below ground, plant roots can interact with soil microbes to form unique rhizosphere microbial communities that respond to environmental conditions (Pascale et al., 2019). Plant-driven rhizosphere microbes undergo transformation under drought stress to improve drought resistance for plant growth and development under drought conditions (Zeng et al., 2018). Although the mechanisms associated with plant responses to drought stress have been extensively studied from the perspectives of morphology, physiology and genetics, the effects of drought on plant rhizosphere microbiota are still poorly understood (Munoz-Ucros et al., 2022).

Plants employ a variety of strategies to overcome drought stress, including a combination of stress avoidance and regulation of drought tolerance, depending on their genotype. The interaction between plants and the soil microbiomes that colonize the rhizosphere and root system is considered a key factor in the rapid adaptation of plants to soil environmental stress (Pascale et al., 2019). However, changes in the rhizosphere microbial community under drought stress depend on the influence of the host plant and the surrounding soil (Bazany et al., 2022). Differences in plant varieties (genotypes) can lead to different levels of drought tolerance. For instance, drought-resistant plants have evolved various strategies for adapting to drought compared with water-sensitive plants (Hilker et al., 2016) and they can rapidly activate drought tolerance mechanisms at the physiological and molecular levels in response to drought stress. However, a succession of studies have now suggested that this difference in drought tolerance levels between varieties (genotypes) is partly due to rhizosphere microbial differences (Song Y. et al., 2021). Studies on sugarcane (Liu Q. et al., 2021), tomato (Gaete et al., 2021), cotton (Ullah et al., 2019), soybean (Kuzmicheva et al., 2017), rice (Santos-Medellin et al., 2017), and switchgrass (Liu T. Y. et al., 2021) have all supported this view. Host plants are able to alter rhizosphere microbial composition by influencing root structure and secretions (Wen et al., 2020), which, in turn, enhances plant resistance through rhizosphere interactions, although this effect varies widely among species and genotypes. In rice, differences between the microbial communities recruited by indica and japonica rice in the same soil resulted in differences in N use efficiency, and this process was regulated by the NRBT.1 gene (Zhang et al., 2019). However, quantifying the effects of various factors on rhizosphere microbes under drought stress is complex and needs to be further explored.

Alfalfa (*Medicago sativa* L.) is a globally important, widely cultivated leguminous forage crop due to its high biomass production and nutritional value for livestock (Annicchiarico et al., 2015). In China, alfalfa is generally grown in the arid northern regions. With global climate change and the scarcity of water resources, alfalfa cultivation is facing serious challenges. Extreme water shortages can lead to significant yield reductions and reduced quality of alfalfa. Most of current research focuses on improving alfalfa yields and breeding varieties resistant to abiotic stresses (Shi et al., 2017). However, there is still a large knowledge gap about the potential functions of alfalfa rhizosphere microbes in improving its resistance, which has been explored in other plants (Zhao et al., 2020). For example, different core communities of rhizosphere microbiomes constructed from unique varieties of plants such as tomato (Adedayo et al., 2022), citrus (Penyalver et al., 2022), and wheat (Azarbad et al., 2018) were able to improve their environmental adaptability. Similarly, genetically diverse rice varieties respond to drought stress by using specific rhizosphere microbial communities (Santos-Medellin et al., 2017). Currently, most studies of plant-associated microbial communities have been conducted by means of ribosomal amplicon-based approaches (Glick and Gamalero, 2021). Certainly, the advent of metagenomic can further provide taxonomic, genomic, and functional information for a given community and provide reliable technical support for determining the functional mechanisms mediating plant–microbiome interactions and defining the core microbiome of plant species (Xu et al., 2021).

As the second genome of plants, microbiomes have great potential to improve plant resistance. One future direction is to take rhizosphere microbiomes as one of the breeding objectives, and improve stress resistance and yield by transforming plant roots and recruiting beneficial microbiomes through rhizosphere secretions (Mavrodi et al., 2021; Sun et al., 2021). To achieve this, of course, a lot of groundwork still needs to be done. We hypothesized that the diversity of the rhizosphere microbial communities of different alfalfa varieties is the result of long-term selection and shaping of the rhizosphere environment by alfalfa, and that differences in its rhizosphere microbes are one of the important factors contributing to varietal differences in resistance to stress. Therefore, the aim of this study was to analyze the main changes in microbial diversity in the rhizosphere of alfalfa under drought stress. Our aim was not only to demonstrate that soil microbiomes can play a role in plant resistance to drought stress, but also to understand which bacterial communities are specific to the rhizosphere of alfalfa plants with varying drought tolerance. To this end, we selected the drought-tolerant alfalfa variety “Aohan (AH)” and the drought-sensitive alfalfa variety “Stockpile (QS)” and studied their microbial communities under well-watered and drought conditions. Our main research questions were: (1) Can soil microbiomes act to improve the drought tolerance of alfalfa in the face of drought stress?, (2) What are the specific bacterial communities recruited between the rhizosphere of different drought-tolerant alfalfa varieties?, and (3) What are the response pathways of drought-tolerant bacteria in alfalfa rhizosphere under drought stress?

## Materials and methods

### Plant materials and soil

In this study, the drought tolerance performance of eight alfalfa varieties commonly used in production in northern China was compared through preliminary pre-experiments (Supplementary Table 1). By observing the phenotype of alfalfa after sustained drought stress (experimental methods and conditions were the same as in this study), one relatively drought-resistant variety, Aohan (AH), and one relatively drought-sensitive variety, Stockpile (QS), were selected for use in this study (Supplementary Figure 1).

We collected field soil from the experimental site of Inner Mongolia Agricultural University (Hohhot, China) (111 ° 22 ´ 30 “E, N40 ° 41 ´ 30” N), which had not been planted or fertilized, and transported it back to the laboratory for air drying. The air-dried soil was sieved through a 2 mm mesh to remove the debris and large clods, and stored at 4°C.

### Plant germination, transplanting, and cultivation in the greenhouse

AH and QS seeds were first sandpapered and then rinsed with sterile water to break up the seed coat. Surface sterilization was then carried out, by treating seed surface with 4% NaClO for 5 min, and washing it with sterile water six times. Sterilized seeds were placed in a 9 cm Petri dish (two layers of sterile filter paper on the bottom and 5 ml of sterile water). The dish was placed in dark culture at 4°C for 2d, followed by dark culture at 25°C for 2d, then removed and placed at room temperature for 1d. The soil substrates were divided into sterilized (M) and unsterilized (W) treatments. Specifically, vermiculite, volcanic stone, and some field soil were sterilized twice by autoclaving for 30 min. 150 g of volcanic stone and 75 g of vermiculite were weighed separately in 10 × 10 cm pots and the sterilized and unsterilized treatments were defined by adding 25 g of sterilized or unsterilized soil and mixing thoroughly. We then add 1/2 Hoagland nutrient solution to 100% soil water content (250 ml of saturated water content). Seedlings of AH and QS were selected at the same germination status and transplanted into pots containing sterilized and unsterilized soil, with five plants per pot, and 16 pots per treatment. Also, we used pots without transplanted seedlings as bulk soil (BS) controls. The seedlings were grown in an artificial climate chamber under 16/8 h light/dark and 25°C/22°C day/night with approximately 60% relative humidity, and watered with sterilized water every 2 d to 70% saturated water content.

### Drought stress treatment and sample collection

Healthy 4-week-old alfalfa seedlings of approximately uniform growth were selected for the experiment. The sterilized and unsterilized treatments of AH and QS were divided into two groups, one with normal watering (AHMCK, AHWCK, QSMCK, and QSWCK) and one with continuous drought (AHMDr, AHWDr, QSMDr, and QSWDr), with eight pots per treatment as replicates. During the growing period, we used the weighing method to irrigate with sterile water every day. The well-watered group was maintained at 70% saturated water content during the growth period by daily irrigation, while drought stress was applied to the treated group by withholding irrigation until the end of the experiment. Every 2–3 days the pots were randomly rearranged to eliminate the effect of location on the experiment. We measured the soil water content of each treatment every day, and measured the plant height of each alfalfa seedling. During the drought period, plants gradually consumed water and nutrient reserves in the matrix, which simulated field conditions, and no visual symptoms of nutrient deficiency were observed. Sampling was carried out when the plants were observed to show signs of wilting. The chlorophyll fluorescence parameter (Fv/Fm) was measured using a Handy PEA (Hansatech, England). Three pots were selected to measure the height, leaf length and width of each plant, then the aboveground part of each plant was cut off from the base and immediately weighed for fresh weight, after which it was dried in an oven at 65°C for 48 h and the dry weight was measured. At the same time, rhizosphere soil was collected and the rhizosphere soil was separated from the roots of the alfalfa as previously described (Schmitz et al., 2022). In brief, we carefully removed the roots from each pot, then shook them manually to remove the bulk soil. The roots were washed in sterile phosphate buffer (6.33 g NaH_2_PO_4_.H_2_O, 16.5 g Na_2_HPO_4_.7H_2_O, and 200 μl Silwet L-77 in 1 l ddH_2_O, pH = 7.0) and vortexed briefly. After removing the roots, the suspension was filtered through a 100 μm cell strainer and spun down for 10 min at 4000 x g. The supernatant was discarded and the remaining pellet was the rhizosphere. All samples were frozen in liquid nitrogen and stored at −80°C. Leaves from the remaining replicate pots were collected for immediate determination of relative conductivity and chlorophyll content, and the remainder were snap-frozen in liquid nitrogen and placed at −80°C for physiological index determination. The roots were also removed to determine root length, fresh weight, and dry weight.

### Measurement of plant phenotypic and physiological features

Plant height was measured from the base of the shoot to the tip of the tallest leaf. Main root length was measured from the base of the shoot to the end of the root, and leaf area was calculated using the formula: leaf area = leaf length (leaf base notch to leaf tip) × leaf width (the widest part of the leaf) × 0.87. Each treatment of the above indexes had 15 replicates. Leaf water content (LWC) was measured by taking 5–10 leaves from the top of the plant, immediately determining the fresh weight, and then placing them in an 80 ° C oven for 16 h to measure their dry weight. The LWC was calculated with the following equation: LWC = (fresh weight – dry weight)/fresh weight × 100 (Song X. Y. et al., 2021). Relative electrical conductivity (REC) was analyzed as described by Quan et al. (2015). The detached leaves were placed in 50 ml tubes containing 15 ml ddH_2_O and gently shanked for 6 h at room temperature. Then, the leaves were boiled at 100°C for 20 min. When the leaves were cooled to room temperature, measured by the formula REC (%) = (C_i_ /C_max_) × 100, where C_i_ and C_max_, respectively, represent the conductivity before and after boiling of the detached leaves. Chlorophyll fluorescence parameters of leaves representing the maximum photochemical efficiency of photosystem II (PSII) (Fv/Fm) were measured with a Handy PEA (Hansatech, England). Leaves were incubated in the dark for 30 min before fluorescence measurements. Chlorophyll content was determined by spectrophotometry, as described by Jiao et al. (2017). Chl in leaves was extracted with 80% acetone The extract was centrifuged for 10 min at 5300 × g and the supernatant liquid was used to test absorbance under 645 mm and 663 mm wavelengths, respectively. The level of lipid peroxidation, an indicator of cellular damage, was determined from the measurement of malondialdehyde (MDA) content resulting from thiobarbituric acid reaction, as described by Christou et al. (2013). 0.5 g sample was added 5 ml TCA and ground them into homogenate. After centrifugation, removed supernatant, added 2 ml TBA, mixed, bath at 95°C for 25 min, and then cooled to room temperature, reading at a wavelength of 450, 532, and 600 nm. Free proline content was determined using the ninhydrin reaction according to the method of Bates et al. (1973). Proline was extracted with 3% (w/v) sulfosalicylic acid, and the extractions were injected to the compounds of ninhydrin reagent and glacial acetic acid. Then, the mixture was boiled at 100°C for 40 min. When it cooled to the room temperature, the proline content was assayed through the absorbance of 520 nm and determined from a proline standard curve. Soluble protein concentration was determined according to Bradford (1976) using bovine serum albumin as a standard. 100 mg fresh leaves were ground well in 10 ml of 50 mM cooled phosphate buffer (pH 7.8). The homogenate was centrifuged at 6000 × g for 20 min at 4°C. The supernatant was used to determine the total soluble proteins. Catalase (CAT) activity was assayed by monitoring H_2_O_2_ reduction by following the methodology of Maehly and Chance (1954). The reaction mixture consisted of 50 mM potassium phosphate buffer (pH 7), 10 mM H_2_O_2_, and 150 μl enzyme extract to a final volume of 1.5 ml. Each treatment of the above indexes was repeated three times.

### DNA extraction, PCR amplification, sequencing, and raw data analysis

DNA from soil and rhizosphere samples was isolated using the PowerSoil DNA Isolation Kit (Mobio Laboratories, United States) according to the manufacturer’s instructions. The concentration and quality of the DNA were evaluated with a NanoDrop 2000 spectrophotometer (Thermo Fisher Scientific, MA, United States). The V3–V4 region of the 16S rRNA gene was amplified using the universal primers (341F: 5′ -CCTACGGGNBGCASCAG-3′ and 806R: 5′ -GACTACNVGGGTATCTAATCC-3′) (He et al., 2021). Every 50 μl PCR reaction contained 2 μl of diluted DNA, 25 μl of 2X Phanta Max Master Mix (Vazyme P515-01, China), 10 pM of forward and reverse primers, and 21 μl of Nuclease-Free Water (PROMEGA). The thermal cycling consisted of initial denaturation at 95°C for 30 s, followed by 28 cycles of 95°C for 15 s, 55°C for 15 s and, 72°C for 30 s, and finally at 72°C for 5 min (Li et al., 2022). The PCR products were purified using the QIA quick Gel Extraction Kit (QIAGEN Germany) according to manufacturer’s instructions and quantified using Quantus™ Fluorometer (Promega, United States). The sequencing library was generated using the KAPA Library Preparation Kit (Kapa, MA, United States) by following the manufacturer’s instructions and was quantified with an Agilent Bioanalyzer 2100 system. Finally, the quantified library was sequenced on the NovaSeq 6000 platform (Illumina, CA, United States). Raw reads were firstly filtered by Trimmomatic (v.0.33). Reads containing N or quality score below 20 or with a length < 50 bp were discarded. Then the primer sequences were identified and removed by Cutadapt (v.1.9.1), which finally generated high-quality reads without primer sequences. Paired-end high-quality reads were merged using FLASH (v.1.2.11) based on the overlap between these reads, which generated clean reads. The minimum length of overlap was set to 10 bp, and the maximum allowable error ratio of the overlap region was 0.1. A total of 1,436,895 high-quality clean reads were obtained from the replicated samples in 6 groups. De-noise was processed by DADA2 (Callahan et al., 2016) in QIIME2 in order to remove chimeric sequences, generating ASVs. Conservative threshold for ASVs filtration is 0.005%. Chloroplast sequences in all samples were removed. To mitigate the impact of the inconsistent sequences, all samples were rarefied to 20,000 sequences for analysis. Taxonomy analysis of ASVs based on the Sliva 16S rRNA gene database (v.138) using the classify-sklearn algorithm with the Naive bayes classifier in QIIME2.

### Statistical and bioinformatics analyses

Statistical analysis was performed with SPSS software (v.26.0.0.2). Student’s *t*-test were used for significance analysis between different groups (*p < 0.05, **p < 0.01, and ***p < 0.001 were considered to be significant). Bar graphs were plotted with Prism 8.4.3 (GraphPad Software, LLC). The alpha diversity of each sample was analyzed based on four alpha diversity indices: Chao1, Shannon, Simpson, and Ace. All sample indexes were calculated using QIIME (v.1.9.1) and *t*-tests were used to evaluate the differences in alpha diversity index between different treatments. Principal Coordinate Analysis (PCoA) based on weighted_unifrac distance was performed with the R Package vegan (v.2.5.7). A histogram of species distribution and a heat map were generated with the ggplot2 (v.3.3.5) package. Linear discriminant analysis effect size (Lefse), for which the logarithmic LDA score was set to 4.0 with statistical significance (*p* < 0.05), and the functional potential of a bacterial community predicted by PICRUSt2 were both performed on the BMK Cloud platform.^1^

## Results

### Phenotypic response of soil microbiomes to improve drought tolerance of alfalfa

**Figure 1:**
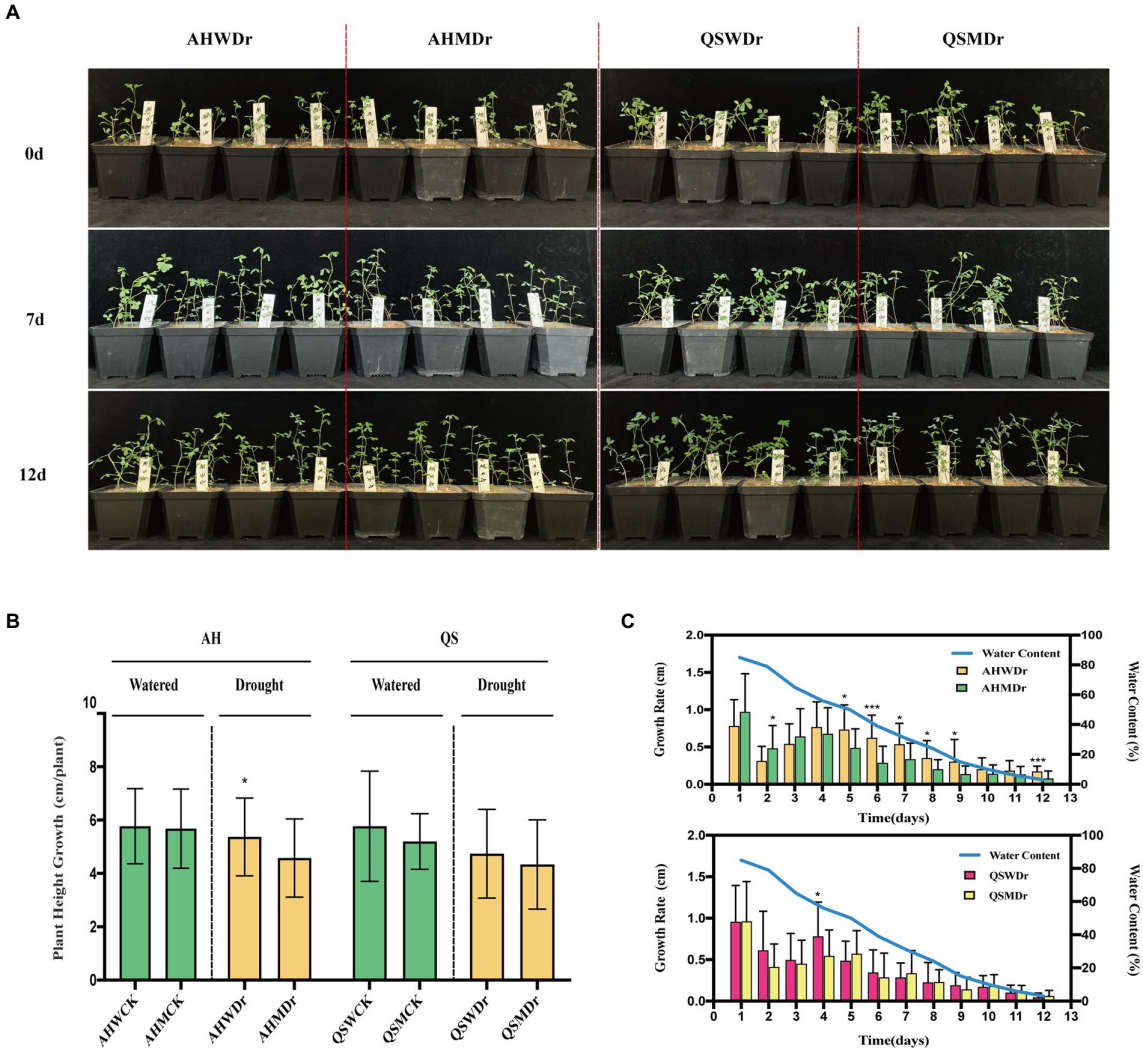
Effects of soil microbiomes on growth phenotype, plant height growth, and growth rate of alfalfa. **(A)** Photographs of growth phenotypes of two treatments (sterilized and unsterilized) and two varieties (AH and QS) during drought stress, **(B)** Plant height growth of AH and QS in sterilized and unsterilized soil under well-watered and drought stress conditions from day 0–12, and **(C)** Changes in growth rate of AH and QS in sterilized and unsterilized soil during drought stress. Student’s *t*-test was used to analysis the significance difference of sterilized and unsterilized on plant height growth and growth rate of the two varieties under well-watered and drought stress. Data are the means of 15 replicates, and error bars indicate standard deviations. Asterisks indicate statistical significance, *p < 0.05, **p < 0.01, and ***p < 0.001.

We found that the unsterilized treatment significantly increased the drought tolerance of alfalfa and improved the growth of alfalfa under drought stress compared to the sterilized treatment. Interestingly, soil microbiomes were more effective for the drought-tolerant variety AH (Figure 1A). Specifically, there was no significant difference in relative plant height increase, leaf area, main root length, and fresh weight or dry weight of aboveground and underground parts between the sterilized and unsterilized alfalfa varieties under well-watered conditions. However, under drought stress, these indexes for unsterilized AH were significantly higher than those for sterilized AH. The plant height growth of AHWDr (5.48 cm) significantly higher than that of AHMDr (4.55 cm) (Figure 1B). With a continuous decrease in water content (Supplementary Figure 2, Supplementary Table 2), the growth rate of AHMDr was higher than that of AHWDr in the first 3 days, but from day 5 onward, that of AHWDr was significantly higher than that of AHMDr. However, the growth rate of QSWDr was significantly higher than QSMDr only on day 4, with no significant differences at any other days (Figure 1C).

**Figure 2:**
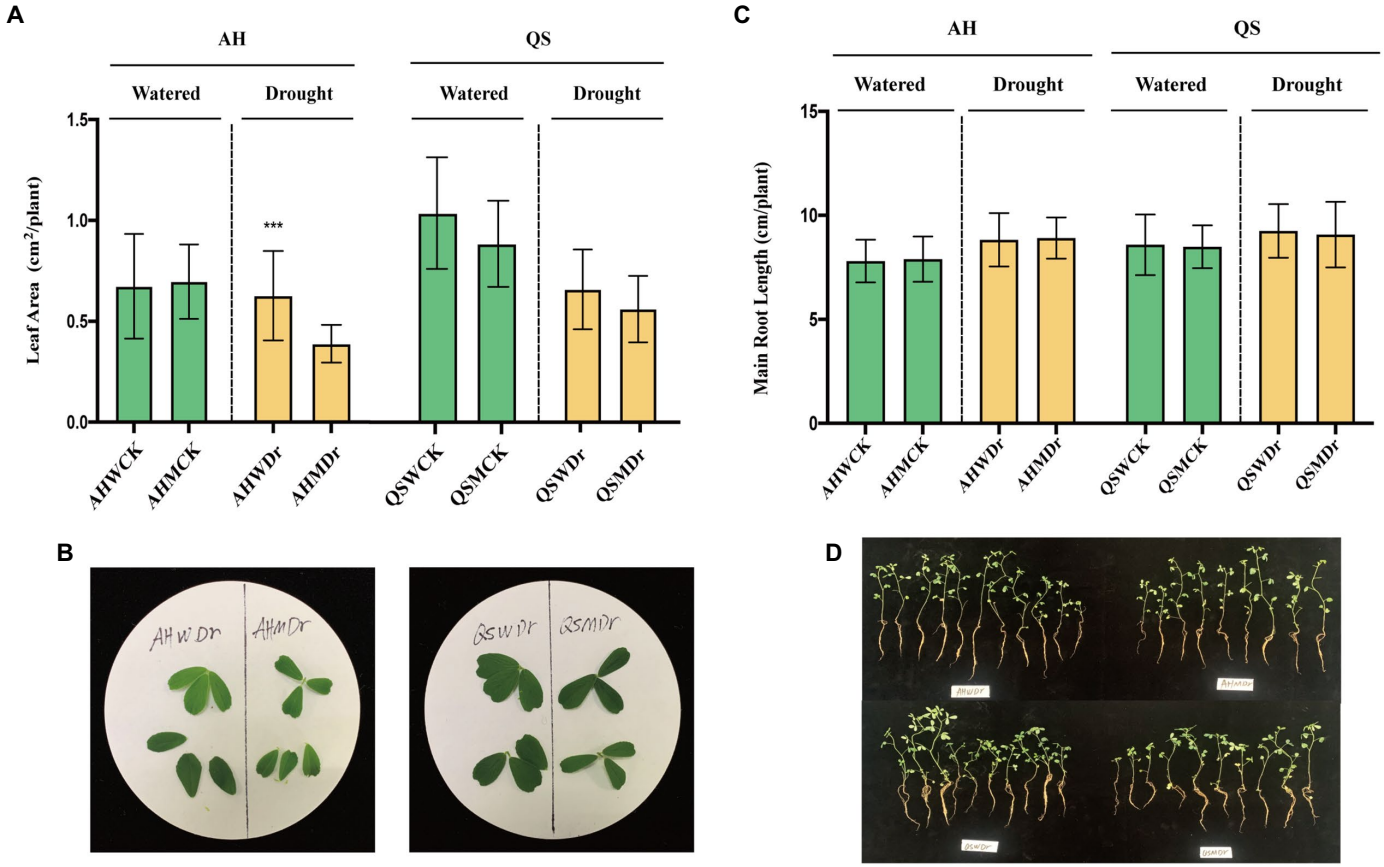
Effects of soil microbiomes on leaf area and main root length. **(A)** Leaf area of AH and QS in sterilized and unsterilized soil under well-watered and drought stress conditions, **(B)** Leaf photographs of AH and QS in sterilized and unsterilized soils under drought stress, **(C)** Main root length of AH and QS in sterilized and unsterilized soil under well-watered and drought stress conditions, and **(D)** Main root photographs of AH and QS in sterilized and unsterilized soils under drought stress. Student’s *t*-test was used to analysis the significance difference of sterilized and unsterilized on leaf area and main root length of the two varieties under well-watered and drought stress. Data are the means of 15 replicates, and error bars indicate standard deviations. Asterisks indicate statistical significance, *p < 0.05, **p < 0.01, and ***p < 0.001.

**Figure 3:**
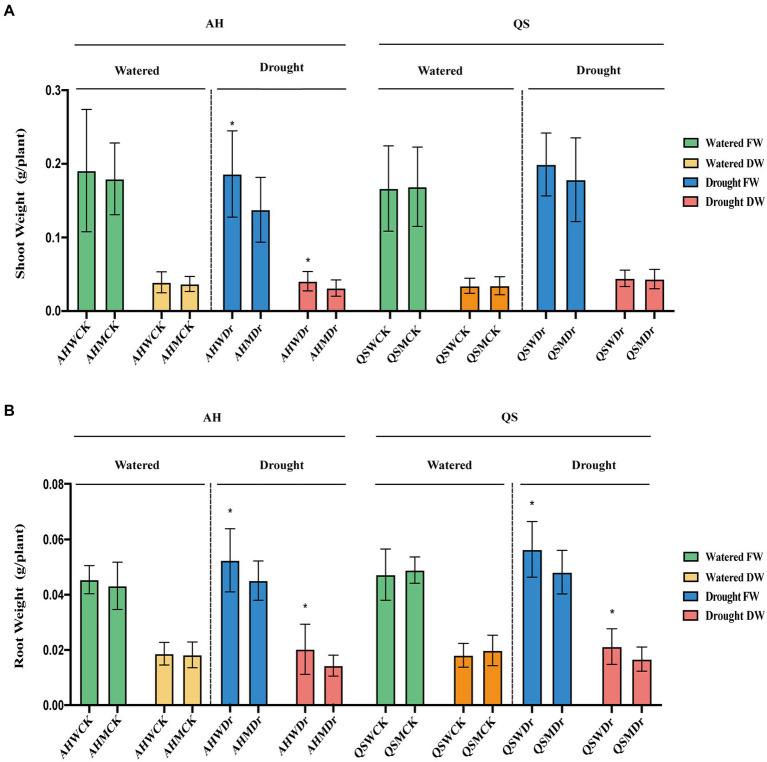
Effects of soil microbiomes on above- and belowground biomass. **(A)** Fresh and dry weights of AH and QS shoot in sterilized and unsterilized soil under well-watered and drought stress conditions, **(B)** Fresh and dry weights of AH and QS roots in sterilized and unsterilized soil under well-watered and drought stress conditions. Student’s *t*-test was used to analysis the significance difference of sterilized and unsterilized on above- and belowground biomass of the two varieties under well-watered and drought stress. Data are the means of 15 replicates, and error bars indicate standard deviations. Asterisks indicate statistical significance, *p < 0.05, **p < 0.01, and ***p < 0.001.

We found that drought stress significantly reduced the leaf area of alfalfa under sterilized conditions, but soil microbiomes could effectively improve this situation, with AHWDr having a significantly higher leaf area than AHMDr (Figures 2A,B). Drought stress resulted in higher length of the main roots of alfalfa than the well-watered treatment, but microbial effects on main root length were not significant (Figures 2C,D). We also measured the biomass of above- and belowground parts. Although soil microbiomes did not increase the biomass of aboveground parts of alfalfa under well-watered conditions, the aboveground biomass of the unsterilized treatment was higher than that of the sterilized treatment during drought stress, with only 136.17 and 31.03 mg fresh and dry weight for AHMDr compared to 184.15 and 40.04 mg for AHWDr (Figure 3A), which were higher than AHMDr by 35.24 and 29.04%, respectively. However, the microbial improvement in QS was not significant. For belowground plant parts, there were also no significant differences under well-watered conditions, but microbiomes significantly increased both fresh and dry weights of the belowground parts under drought stress (Figure 3B). Our results show that soil microbiomes can significantly improve the drought tolerance of alfalfa and its growth status under drought conditions, but this effect is variety-specific, with soil microbiomes being significantly more effective on AH than on QS.

### Physiological and biochemical responses of soil microbiomes to improve drought tolerance in alfalfa

**Figure 4:**
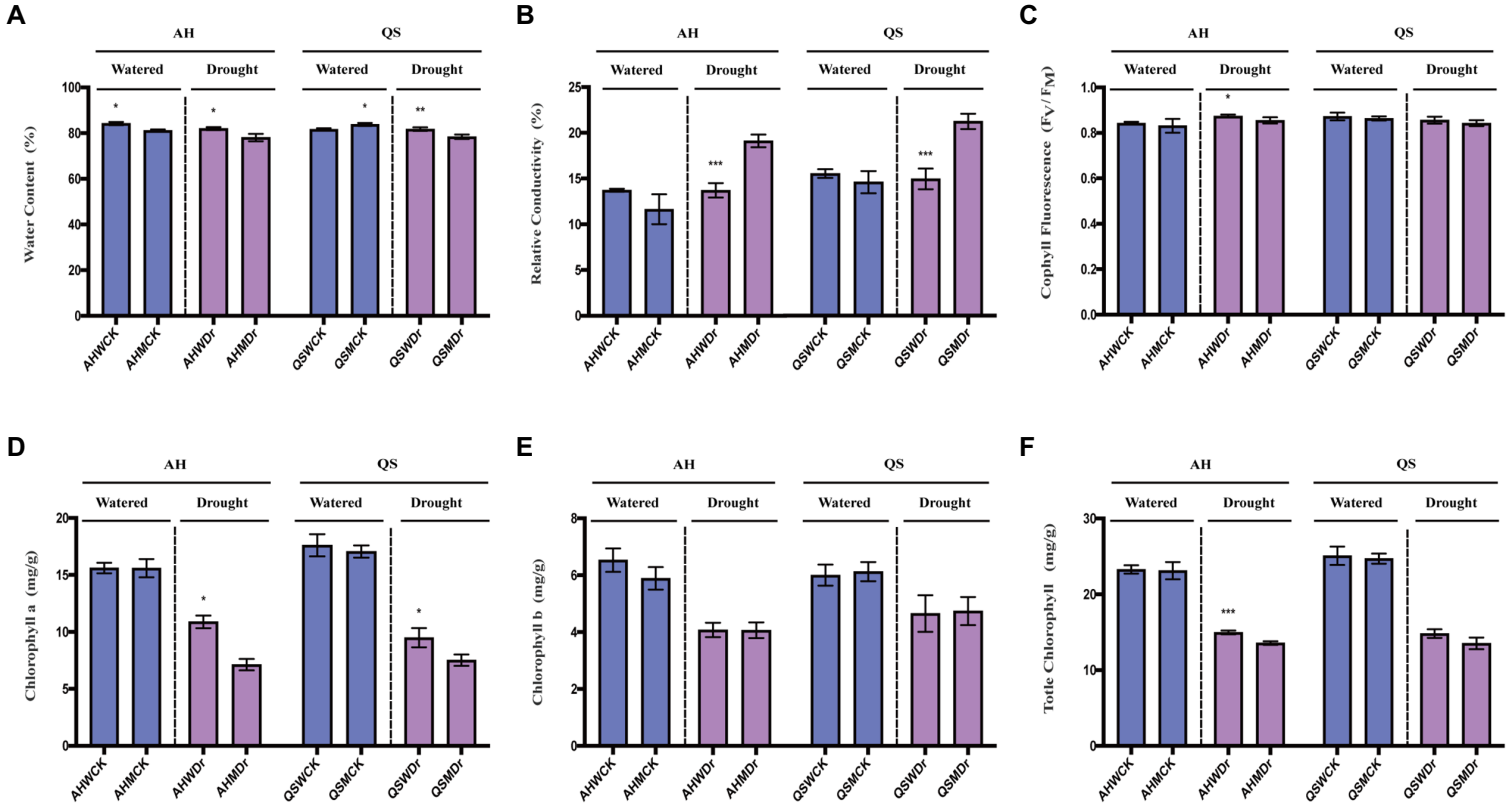
Effects of soil microbiomes on physiological characteristics. **(A)** Leaf water content, LWC, **(B)** Relative electrical conductivity, REC, **(C)** Chlorophyll fluorescence parameters, Fv/Fm, **(D)** Chlorophyll a, Chl_a_, **(E)** Chlorophyll b, Chl_b_, and **(F)** Total Chlorophyll, Chl_t_. Student’s *t*-test was used to analysis the significance difference of sterilized and unsterilized on physiological characteristics of the two varieties under well-watered and drought stress. Data are the means of 15 replicates, and error bars indicate standard deviations. Asterisks indicate statistical significance, *p < 0.05, **p < 0.01, and ***p < 0.001.

We further analyzed the role of soil microbiomes in improving drought tolerance in alfalfa at the level of physiological characteristics and the differences in physiological and biochemical indicators of different alfalfa varieties. Leaf water content (LWC) is one of the most important indicators of the extent to which plants are affected by drought. We found that microbiomes significantly increased the LWC of AH under well-watered and drought conditions, while LWC of QS only increased under drought conditions and was even lower than the sterilized treatment under well-watered conditions (Figure 4A). REC of the leaves can characterize the extent of damage to plant cell membranes. Drought led to significant increase in REC, but REC of both AH and QS in the unsterilized treatment was extremely significantly lower than those of the sterilized treatment (Figure 4B). Microbiomes were able to help AH maintain a higher maximum photochemical efficiency (Fv/Fm) during drought stress, while this effect was not evident on QS (Figure 4C). We also measured the chlorophyll content. Under well-watered conditions, microbiomes had no significant effect on Chl_a_, Chl_b_, or Chl_t_ content. Drought stress inhibited the synthesis of chlorophyll content, but the microbiomes were able to significantly increase the Chl_a_ content of AH and QS during drought compared to the sterilized treatment (Figure 4D), and were able to increase the Chl_t_ content of AH extremely significantly (Figure 4F), with no significant effect on Chl_b_ (Figure 4E).

**Figure 5:**
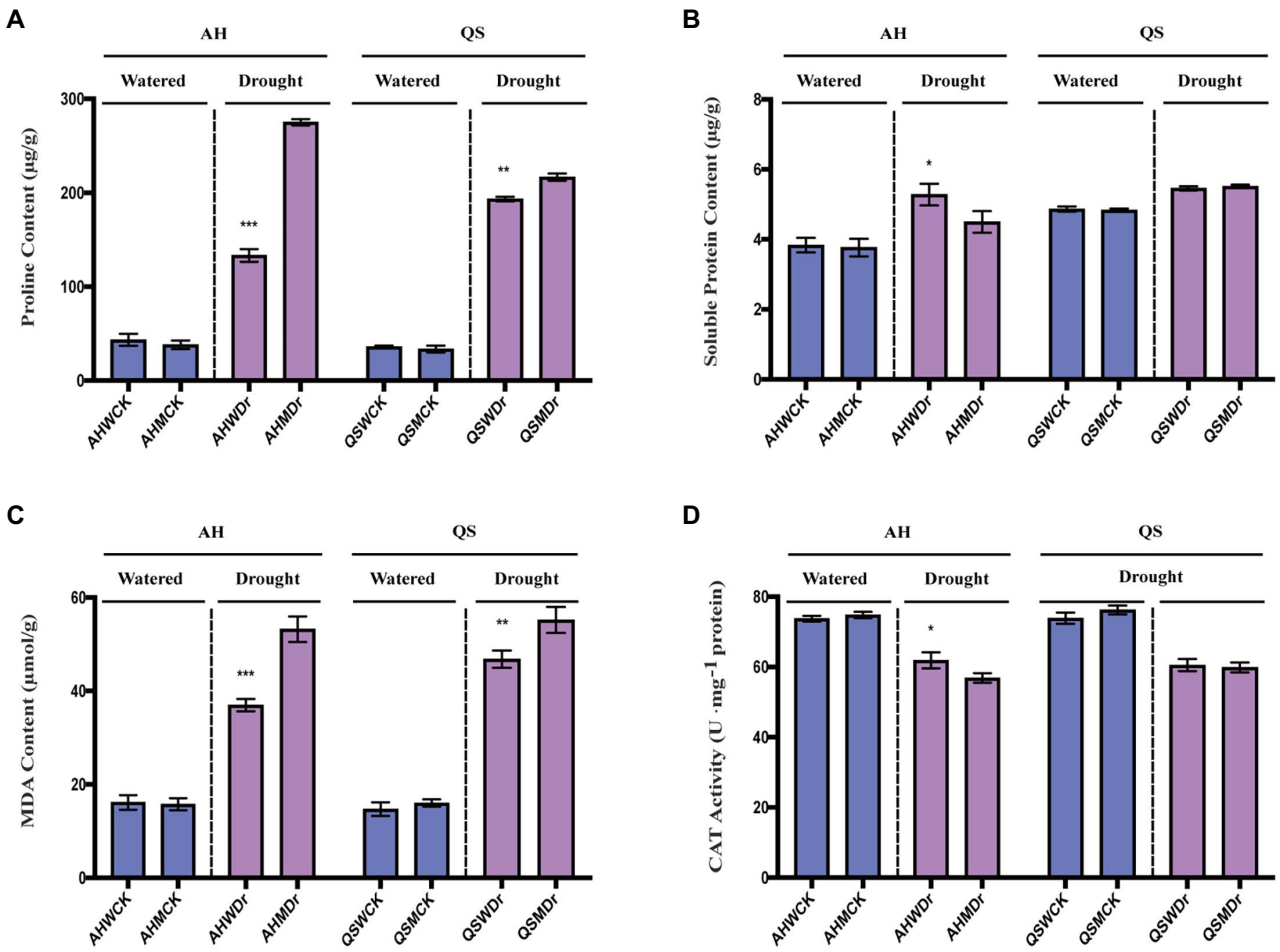
Effects of soil microbiomes on biochemical indicators. **(A)** Proline content, **(B)** Soluble protein, **(C)** MDA content, and **(D)** CAT activity. Student’s *t*-test was used to analysis the significance difference of sterilized and unsterilized on biochemical indicators of the two varieties under well-watered and drought stress. Data are the means of 15 replicates, and error bars indicate standard deviations. Asterisks indicate statistical significance, *p < 0.05, **p < 0.01, and ***p < 0.001.

Under well-watered conditions, the proline content of both AH and QS was low and did not differ significantly between the bacterial and sterile treatments. After drought stress, the proline content of both varieties increased significantly, and interestingly, the increase in proline was very significantly lower in both the AH and QS bacterial treatments than in the sterile treatment (Figure 5A). Soluble proteins are important osmoregulatory substances and their increase and accumulation improve the water retention capacity of cells. We found that microbiomes can also help AH to significantly increase its soluble protein content during drought (Figure 5B). MDA is a product of oxidation of plant cell membrane systems and is a marker of plant exposure to adverse stress. We found that drought stress significantly increased MDA content of alfalfa, but the increase in MDA for both AH and QS in the unsterilized treatment was extremely significantly lower than in the sterile treatment (Figure 5C). CAT activity is related to the metabolic intensity of the plants, and drought stress inhibits plant metabolism. Our results showed that drought stress reduced the CAT activity of both alfalfa varieties, but the reduction for AH in the unsterilized treatment was relatively small, and its activity was significantly higher than that of the sterilized treatment (Figure 5D). In summary, our results illustrated that microbiomes can help alfalfa to effectively mitigate the drought stress to which it is subjected by reducing the degree of damage to cell membranes and increasing metabolic enzyme activity, thereby improving drought tolerance in alfalfa. However, this effect is variety-specific, with soil microbiomes having a more significant effect on AH than QS.

### Effects of drought on the diversity of rhizosphere microbial communities of alfalfa

**Figure 6:**
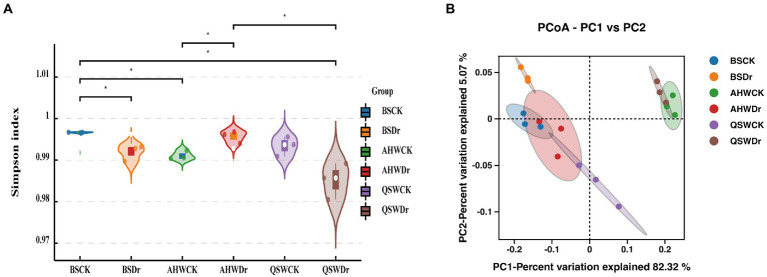
Analysis of differences in microbial diversity based on water treatment and variety differences. **(A)** The Shannon index based on *t*-test represents the variation of alpha diversity in each treatment and **(B)** PCoA analysis (for principal coordinates PCo1 and PCo2) of beta diversity based on weighted_unifrac distance shows that the different water treatments and varieties were separated in the area on the abscissa.

We have found that microbiomes play a key role in alfalfa in the face of drought and have a greater effect on AH than QS. To further explore rhizosphere microbial differences in different varieties of alfalfa in response to drought stress, we collected rhizosphere soil samples from two alfalfa varieties under well-watered or drought conditions, along with bulk soil. The rhizosphere bacterial composition of different alfalfa varieties under well-watered and drought stress conditions was analyzed by sequencing 16S rRNA amplicons on the Illumina NovaSeq 6000p platform. The Shannon index was used to analyze the alpha diversity of the rhizosphere bacterial community of alfalfa under different treatments (Figure 6A). The results showed that there were significant differences in rhizosphere bacterial diversity between bulk soil, AH, and QS under well-watered and drought stress treatments. The differences between BS and QS were not significant under well-watered conditions. Under drought conditions, the differences between BS and AH were not significant. However, there were extremely significant differences in rhizosphere bacterial abundance between AH and QS under drought stress. ACE, Chao1, and Simpson indexes also showed the same results (Supplementary Table 3).

Principal coordinate analysis (PCoA) of weighted_unifrac distance was performed to reveal the overall dissimilarity of rhizosphere bacterial compositions (Figure 6B). The samples of each treatment were gathered together, but the community distribution of the treatment was scattered. BS was less affected by drought, but AH and QS were more affected by drought stress, and the communities under well-watered and drought stress treatments of AH and QS were distributed on both sides of the first principal coordinate (PCo1), with a significant difference. The communities under AH and QS drought stress were also distributed on both sides of the second principal coordinate (PCo2), indicating that drought and variety had a great impact on the composition of rhizosphere bacterial phylogeny of alfalfa.

### Effects of drought stress on the composition of rhizosphere microbial communities of alfalfa

**Figure 7:**
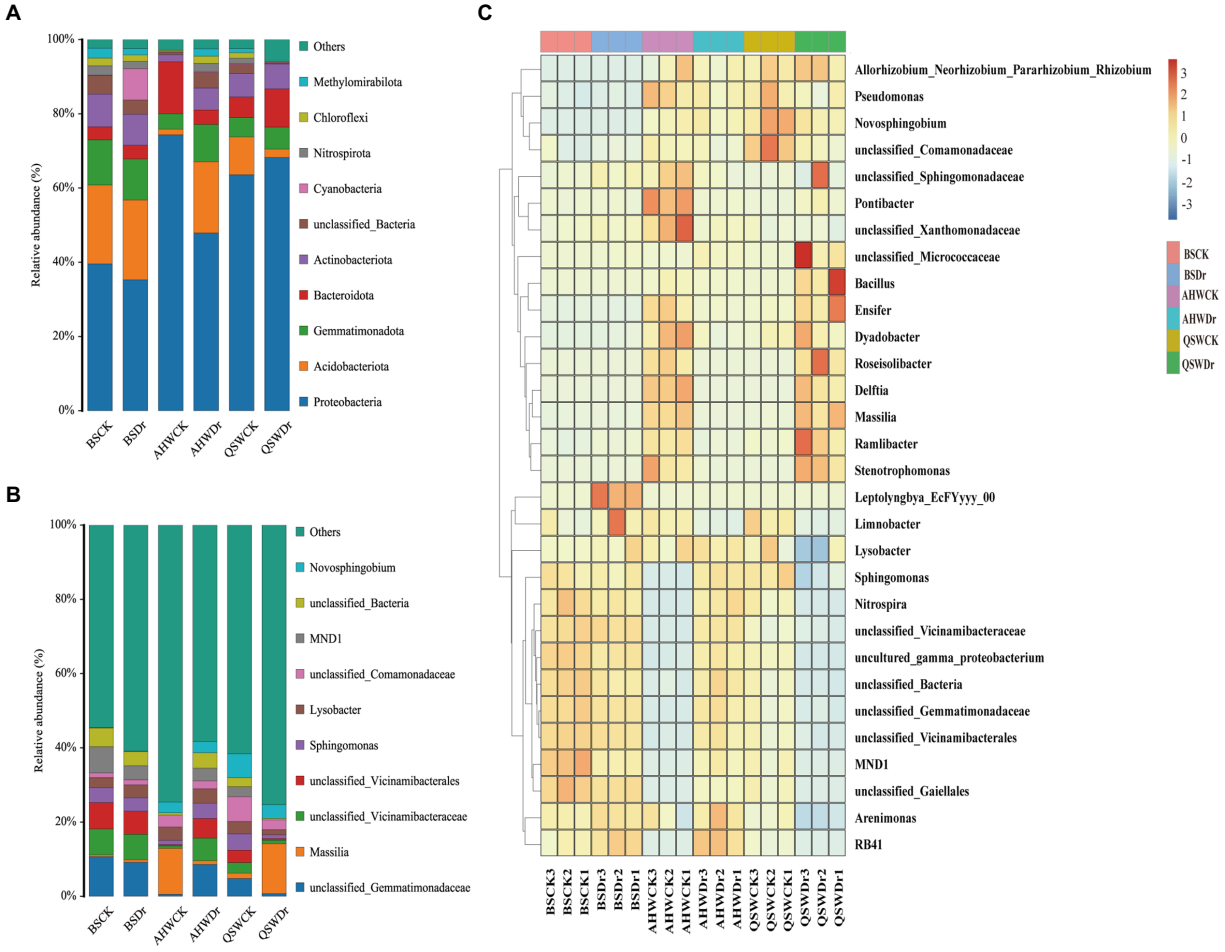
Differences in the composition of bacterial communities in different water conditions and varietal treatments. **(A)** The percentage of the bacterial community (relative abundance top 10) on the phylum level in different water and varietal treatments, **(B)** The percentage of the bacterial community (relative abundance top 10) on the genus level in different water and varietal treatments, and **(C)** A heat map clustering shows the average relative abundance of the top 30 genera of all samples.

A phylum-based population structure analysis of the rhizosphere bacteria under different treatments revealed the dominant microflora in the rhizosphere based on their relative abundance (Figure 7A, Supplementary Table 4). The top three relatively abundant phyla were found to be *Proteobacteria*, *Actinobacteria*, and *Gemmatimonadota*. Drought had a small effect on bacterial community composition and abundance in BS, with only *Cyanobacteria* increasing in abundance. Compared to BS, alfalfa was specifically enriched for certain types of bacteria in soil, and drought, in turn, led to changes in the abundance of various types of bacteria. We found that when AH was well-watered, the rhizosphere significantly enriched *Proteobacteria* (74%) and *Bacteroidetes* (14%), while under drought, the abundance of *Proteobacteria* (48%) and *Bacteroidetes* (4%) decreased, and the abundance of *Acidobacteria* (19%) and *Gemmatimonadota* (10%) increased. However, QS had the opposite trend. Under well-watered conditions, the rhizosphere of QS significantly enriched *Proteobacteria* (64%) and *Acidobacteria* (10%), while under drought the abundance of *Acidobacteria* (2%) decreased, and the abundance of *Proteobacteria* (68%) and *Bacteroides* (10%) increased. Interestingly, the diversity of microbial composition of AH was low under well-watered conditions, but higher under drought conditions. By contrast, QS was high under well-watered and low under drought conditions. Perhaps AH can recruit more microbiomes to help with drought stress, while QS can recruit more microbiomes to promote growth when well-watered.

At the genus level, the top three relatively abundant bacteria were found to be unclassified_ *Gemmatimonadaceae*, *Massilia*, and unclassified_*Vicinamibacteraceae* (Figure 7B, Supplementary Table 5). We found that AHWCK and QSWDr had similar bacterial community composition and relative abundance, and both were quite different from BS. Among them, *Massilia* had the highest abundance, accounting for 12 and 13%, respectively. The bacterial community composition of AHWDr and QSWCK was similar to that of BS, but the abundance was different. The highest abundance bacteria of AHWDr were unclassified_ *Gemmatimonadaceae* (9%), and the highest abundance in QSWCK was unclassified_ *Comamonadaceae* (7%). The top 30 genus from all samples were selected to further investigate the composition of alfalfa rhizosphere bacteria (Supplementary Table 6), and a heatmap of these bacteria was generated (Figure 7C). We can see that there was little difference between bulk soil treatments. AH and QS showed opposite trends in rhizosphere bacteria abundance under well-watered and drought stress conditions. *Massilia*, *Stenotrophomonas*, *Pseudomonas*, *Lysobacter*, *Delftia*, unclassified_*Comamonadaceae*, *Roseisolibacter*, *Pontibacter*, *Novosphingobium*, *Ensifer*, and *Dyadobacter*, whereas very few were found in QS, for which only unclassified_ *Comamonadaceae*, *Novosphingobium*, unclassified_ *Gemmatimonadaceae*, and *Sphingomonas* were relatively more numerous. Under drought stress, AH contained more unclassified_ *Gemmatimonadaceae*, unclassified_ *Vicinamibacteraceae*, unclassified_*Vicinamibacterales*, unclassified_ *Bacteria*, *Sphingomonas*, *Lysobacter*, *MND1*, and *Novosphingobium*, while these were less abundant in QS, which contained more *Massilia*, *Stenotrophomonas*, *Roseisolibacter*, unclassified_ *Micrococcaceae*, *Novosphingobium*, *Bacillus*, and *Ensifer*. Interestingly, we found that *Novosphingobium* was enriched in each treatment, and it may be considered a core microorganism associated with alfalfa.

### Variety-driven differences in rhizosphere drought-tolerant bacteria

**Figure 8:**
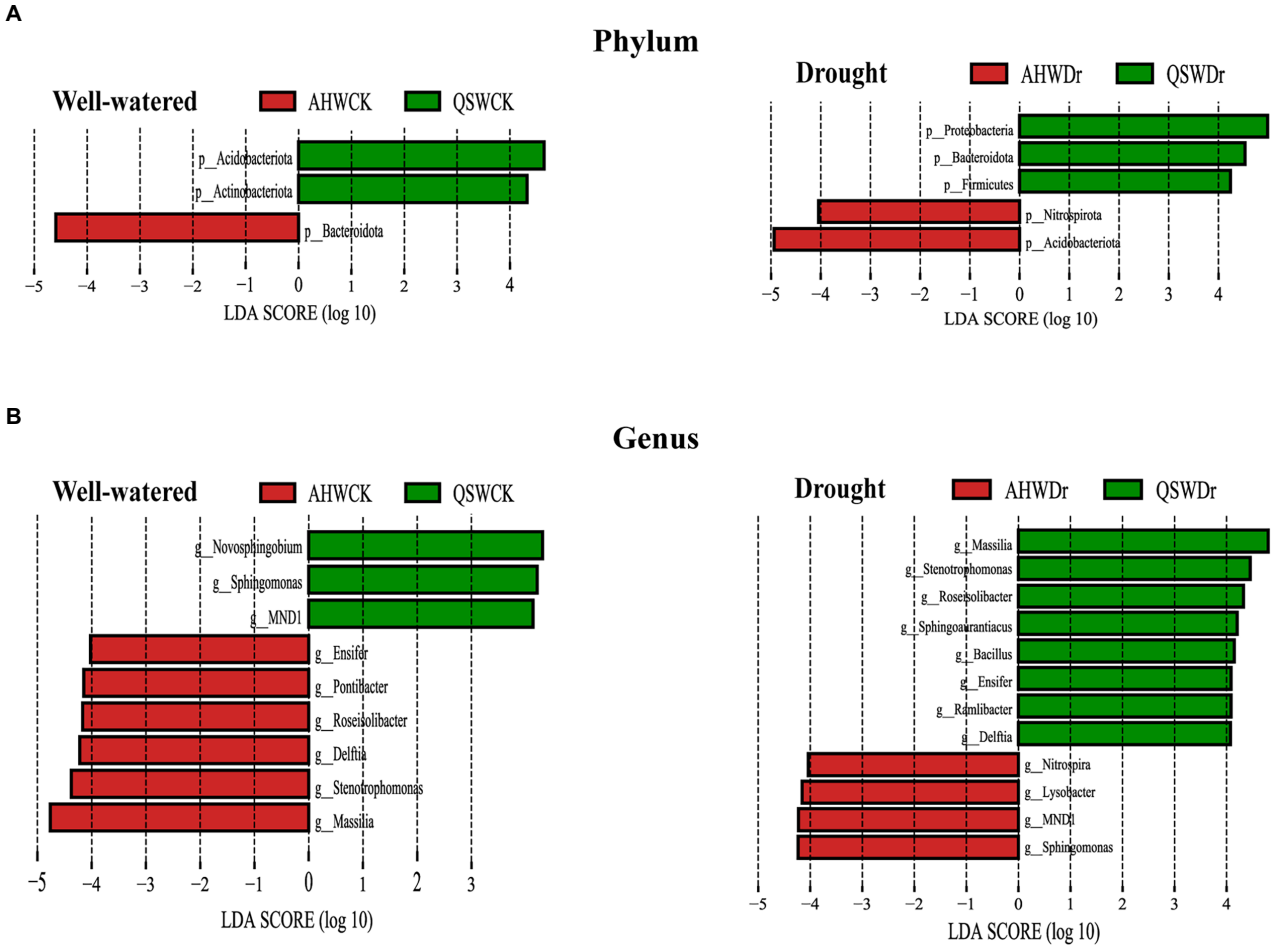
Specific biomarkers of AH and QS rhizosphere under well-watered and drought stress conditions. **(A)** Phylum level biomarkers identified using linear discriminant analysis effect size (LEfSe) analysis using the Kruskal–Wallis test (*p* < 0.05) with linear discriminant analysis (LDA) score > 4.0 and **(B)** Genus level biomarkers identified using linear discriminant analysis effect size (LEfSe) analysis using the Kruskal–Wallis test (*p* < 0.05) with linear discriminant analysis (LDA) score > 4.0.

Although we observed the effect of drought and variety on the shift in abundance of the alfalfa microbiome and identified a group of abundant taxa in rhizosphere soils, biomarkers in rhizosphere soils remain unidentified. Therefore, we sought to identify microbes as biomarkers that most likely explained the observed differences between drought treatments or varieties by performing linear discriminant analysis effect size (LEfSe). In this study, microbes with LDA score > 4 were identified as biomarkers. Results showed that the rhizosphere microbes we identified as biomarkers to alfalfa differed significantly under well-watered and drought stress conditions. Moreover, different varieties had their own specific biomarkers under different water treatment conditions. Specifically, at the phylum level, *Bacteroidota* and *Acidobacteriota* were considered potential biomarkers in well-watered AH and QS rhizosphere soil, respectively. However, after drought stress, *Acidobacteriota* and *Proteobacteria* had high LDA scores in AH and QS rhizosphere soils, respectively (Figure 8A). At the genus level, *Massilia* and *Novosphingobium* were considered potential biomarkers in well-watered AH and QS rhizosphere soil, respectively. However, after drought stress, *Sphingomonas* and *Massilia* had high LDA scores in AH and QS rhizosphere soils, respectively (Figure 8B), pointing to their potential value as novel biomarkers for screening plant growth-promoting rhizobacteria (PGPR) of two alfalfa varieties under drought conditions.

### Functional prediction of rhizosphere bacterial community

**Figure 9:**
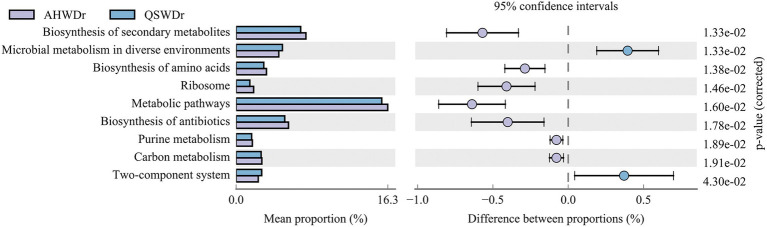
Differences in rhizosphere bacterial KEGG pathways between AH and QS in response to drought stress.

Although our results showed that alfalfa has an increased ability to recruit certain bacteria when facing drought stress and that the recruited bacteria are variety-specific, the functions of these bacteria remain largely unknown. Therefore, we performed a functional prediction analysis of the rhizosphere bacterial communities of the two varieties of alfalfa. The rhizosphere bacterial community differences of the two varieties of alfalfa were exhibited by contrasting the KEGG pathways (Level 3) of the functional abundance TOP10 (Supplementary Table 7). We found that under well-watered conditions, rhizosphere bacteria from AH and QS were significantly enriched in 10 functional pathways including Metabolic pathways, Biosynthesis of secondary metabolites, and Biosynthesis of antibiotics, but these functional pathways were not significantly different between rhizosphere bacteria from AH and QS. However, the rhizosphere bacteria of AH and QS were significantly different in response to drought stress (Figure 9). Rhizosphere bacteria of AH were highly enriched in Biosynthesis of secondary metabolites, Biosynthesis of amino acids, Ribosome, Metabolic pathways, Biosynthesis of antibiotics, Purine metabolism, Carbon metabolism, and were significantly higher than for QS. These pathways suggest that AH rhizosphere bacteria interact more strongly with each other during drought than QS. There is strong compound and energy exchange between them. Notably, metabolic pathways, biosynthesis of secondary metabolites, and biosynthesis of antibodies were the more enriched pathways. We also noticed that other significantly enriched metabolic pathways, such as microbial metabolism in diverse environments and two-component system were more significant in QS rhizosphere bacteria, and these two pathways played more important roles during QS drought stress.

## Discussion

Drought is probably the abiotic stress that has the strongest effect on soil biota (Leng and Hall, 2019). Drought affects plant water potential and swelling, interferes with plant function, and alters plant physiological and morphological characteristics (Wahab et al., 2022). As also found in our results, drought significantly reduced seedling plant height and leaf area. Drought also increases soil heterogeneity, limits the access and flow of phytotoxic nutrients, and increases soil oxygen, often leading to significant decreases in microbial biomass (Naylor and Coleman-Derr, 2018; Jansson and Hofmockel, 2020). Thus, drought can strongly affect both above- and belowground processes. Plants can use soil microbiomes to improve drought tolerance and physiological and biochemical characteristics (Fitzpatrick et al., 2019). However, the contribution of plant-microbiome interactions to drought tolerance in alfalfa remains unclear. In this study, we used an innovative experimental approach to investigate the role of soil microbial communities in improving drought tolerance in alfalfa and to provide new insights into plant drought resistance.

Our first hypothesis was that soil microbiomes play a key role in enhancing drought stress in alfalfa. This was supported by our data. We found that a sustained decrease in water content significantly reduced the relative growth rate and biomass production of alfalfa seedlings, which is consistent with previously reported studies (Anower et al., 2015). However, drought-induced growth inhibition and biomass reduction were significantly lower in unsterilized soil treated alfalfa seedlings under drought stress, and growth performance was better than that of the sterilized treatment (Figures 1–3), as indicated by faster growth rate and larger leaf area, which is consistent with previous observations that plants are enriched with beneficial soil microbiomes to maintain growth when subjected to drought stress (Xi et al., 2018; Bhattacharyya et al., 2021). In other words, high microbial diversity could have stimulated lateral root branching, thus enhancing root prospection in the soil and water perception by roots and leading to a higher stomatal conductance (Prudent et al., 2020). Moreover, some soil microbial species, known as PGPR, secrete compounds capable of altering root structure (Mabood et al., 2014), and can improve water and nitrogen uptake by plant roots under water deficit conditions (Khan et al., 2020). Specifically, drought stress can induce the growth and enrichment of certain gram-negative bacteria to the rhizosphere, promote lateral root growth and main root elongation, increase osmotic pressure, maintain bulking pressure, and protect the macromolecular structure of plant tissues (Schimel et al., 2007). In turn, they delay plant dehydration and maintain plant water status at optimal levels, thus showing high tolerance to water deficit conditions. The physiological and biochemical indices also supported our hypothesis. It has been widely confirmed that rhizosphere bacteria increase the content of leaf water which results in increased plant resistance under drought stress (Cheynier et al., 2013; Durán et al., 2016). At the same time, plants under drought stress maintain lower plasma membrane permeability and stomatal conductance, reduce malondialdehyde content, and promote the synthesis of proline and related hormones, which significantly improves plant drought resistance (Yasmin et al., 2021, 2022). Similar results were found in our study. We observed that unsterilized alfalfa seedlings exhibited higher levels of leaf water content, chlorophyll content, and chlorophyll fluorescence under drought stress conditions (Figure 4). In addition, relative conductivity, proline content, soluble protein content, and lipid peroxidation level (MDA) were lower in unsterilized seedlings compared with sterilized seedlings, suggesting that soil microbiomes enriched at the root interval can alleviate drought stress (Figure 5). Microbiomes were able to effectively maintain water content and provide osmotic protection by regulating soluble protein and proline homeostasis, thus limiting redox damage to cells. All these results demonstrate that soil microbiomes play an important role in improving drought tolerance in alfalfa.

Our second hypothesis was that alfalfa varieties with different drought tolerance would recruit different specific bacterial communities. Again, our data support this hypothesis. 16 s combined with plant growth assays of two alfalfa varieties (drought-resistant AH and drought-sensitive QS) demonstrated that drought-induced rhizosphere bacteria rescue AH and QS from drought challenges. This implies that, despite the importance of genetic differentiation, plants appear to exhibit broadly similar phenotypic and physiological and biochemical response characteristics in the presence of drought. That is, the recruitment of specific root-associated rhizosphere bacteria capable of enhancing plant adaptation to drought stress mitigates the plant’s exposure to drought stress and enables it to sustain growth. However, although drought-induced rhizosphere bacteria could maintain sustained growth in alfalfa, drought-resistant and drought-sensitive varieties recruited different bacterial species and associated functions when challenged by drought, respectively. This means that plants employ variety-specific strategies to recruit beneficial soil bacteria, which can help them solve water challenges. In fact, plants are powerful drivers and selective forces in the evolutionary history of natural microbiomes. For example, the rhizosphere of alfalfa shows a strong preferential enrichment mainly for the three major phyla *Proteobacteria*, *Acidobacteria*, and *Bacteroidetes* (Xiao et al., 2017). Even different varieties have a largely similar species composition. Further, plant adaptation to stress can change the bacterial composition and abundance in the rhizosphere (Kichko et al., 2022), as was confirmed in our experiments. We found large differences between bulk soil and root-associated microbial communities, with reduced relative abundance of rhizosphere bacteria compared to bulk soil (Figure 6), which supports previous findings in crop species (Turner et al., 2013). In addition, we found *Proteobacteria*, *Acidobacteria*, *Gemmatimonadota*, *Bacteroidetes*, and *Actinobacteriota* to be the main bacterial phyla in alfalfa rhizosphere. In particular, *Proteobacteria* and *Bacteroidetes* were also detected in bulk soil with relatively lower abundance compared to rhizosphere samples. Notably, there was an absolute increase in the abundance of *Proteobacteria* at the rhizosphere after the drought treatment, which is consistent with previous studies showing the enrichment of *Aspergillus* under drought stress (Xu et al., 2018; Ullah et al., 2019). *Proteobacteria* are more sensitive to environmental changes because they contain large numbers of non-dormant cells, resulting in increased or decreased abundance (Jones and Lennon, 2010). Interestingly, the relative abundance of *Proteobacteria* and *Bacteroidetes* of drought-sensitive varieties increased significantly under drought stress, while the relative abundance of drought-resistant varieties under well-watered conditions was already high. Exposure to drought stress increased the abundance of *Acidobacteria* in the drought-resistant varieties, while a decrease in abundance occurred at the rhizosphere of the drought-sensitive varieties (Figures 7A, 8A). *Actinobacteriota* was also found to increase in abundance during drought stress in both varieties. We suggest that rhizosphere bacteria with increased relative abundance under drought stress usually have some degree of drought tolerance and can maintain the rhizosphere environment under stress. *Actinobacteriota* is known as a PGPR that promotes the adaptation of host plants under drought stress (Naylor et al., 2017). Our results indicate that under adequate watering conditions, the drought-tolerant alfalfa variety AH already had abundant communities of drought-resistant bacteria, such as *Ensifer* (Ardley, 2017), *Massilia*, and *Stenotrophomonas* (Yang et al., 2021) in the rhizosphere, and that these bacteria began to increase only when the drought-sensitive variety QS was subjected to drought (Figures 7B,C). When drought stress occurred, AH was enriched with more drought-tolerant genera of bacteria such as *Nitrospira* (Xu et al., 2018) and *Sphingomonas*, while the enrichment of this bacteria was weaker in the rhizosphere of QS (Figures 7B, 8B). In addition to the enrichment of some bacteria under normal water conditions, AH was also specifically and significantly enriched with other genera of bacteria, such as *Bacillus* (Hashem et al., 2019). Thus, drought-sensitive varieties require more time to cope with drought stress and are therefore more susceptible to damage. In conclusion, our results demonstrate that alfalfa is enriched with drought-resistant bacteria at the rhizosphere level to help maintain growth when subjected to drought stress and that this effect is variety-specific. That is, alfalfa varieties with different drought tolerance recruit different specific bacterial communities.

So, what are the response pathways and pathways of action of drought-tolerant bacteria in alfalfa roots under drought stress? Numerous studies have shown that plants are strongly associated with their rhizosphere microbiome assembly (Edwards et al., 2015). When plants sense stress, they send distress signals (Rolfe et al., 2019) and then regulate the expression of relevant genes (Zhang et al., 2019) to recruit relevant rhizosphere microbiomes to help alleviate stress by regulating their metabolism and producing various chemical elicitors in the form of root secretions (Stringlis et al., 2018). The amount and composition of exudates varies among plant varieties and genotypes, leading to selective recruitment of associated microbiome members (Naylor and Coleman-Derr, 2018), and these enriched bacterial communities may be important for plants to maintain basic functions. For example, differences in root secretions of major organic components (organic acids, sugars and amino acids) between varieties of soybean lead to specific enrichment and metabolism of rhizosphere bacteria and plant responses (photosynthesis, nodulation, yield, and nutrient uptake) (Kuzmicheva et al., 2017). Our study found that despite the differences between plant varieties and their bacterial taxa, for drought-tolerant or drought-sensitive alfalfa, both *Pseudomonas* and *Novosphingobium* were enriched in AH and QS under drought stress (Figure 7C). This suggests that *Pseudomonas* and *Novosphingobium* have a high potential to enhance the adaptation of alfalfa to drought stress because of their high adaptability to drought environments and affinity for alfalfa roots. In fact, recent studies have shown that most species of *Pseudomonas* can improve plant stress by producing stress-relieving metabolites such as extracellular polysaccharides, gibberellins, ACC deaminases, and indoleacetic acid (Etesami and Glick, 2020). We also noted that *Nitrospira*, *Sphingomonas*, and *Lysobacter* enriched in the rhizosphere. AH has a strong resistance morphology and resistance function, which could inhibit harmful fungi, increase hormone levels, nitrogen fixation, and water retention of the host to improve host resistance (Laborda et al., 2020; Guo et al., 2021). However, *Bacillus* and *Massilia*, which were significantly enriched in QS rhizosphere, can dephosphorylate, induce nutrient uptake and host plant growth, and stimulate host plant defense mechanisms under stress conditions (Sivasakthi et al., 2014; Zheng et al., 2017). For functional prediction of alfalfa rhizosphere bacteria, we found no difference in rhizosphere bacterial function between the two varieties under well-watered conditions. However, under drought stress, the rhizosphere bacterial functions of AH were superior to those of QS in several metabolic-related pathways, including metabolic pathways, biosynthesis of secondary metabolites, biosynthesis of antibodies, biosynthesis of amino acids, and carbon metabolism. Amino acids such as glutamine, proline, and glycine betaine are known to enhance the drought tolerance of plants (Bouskill et al., 2016; Vurukonda et al., 2016). Carbohydrates such as trehalose and raffinose are also known to be drought tolerance enhancers in plants (Santana-Vieira et al., 2016). We speculate that rhizosphere drought-tolerant bacteria can produce a series of secondary metabolites and through these pathways can effectively regulate the C and N content of soil under drought stress, maintain plant C and N balance, promote nutrient uptake and cycling, increase water uptake and retention properties, and improve the rhizosphere environment, which, in turn, can help improve plant drought tolerance (Khan et al., 2020; Poudel et al., 2021). Furthermore, the role of rhizosphere bacteria played a greater role for AH than for QS.

## Conclusion

We investigated the role of soil microbiomes in improving drought tolerance in alfalfa and the changes in the rhizosphere bacteria of two alfalfa varieties with different drought responses. It was demonstrated that soil microbiomes are able to improve drought tolerance in alfalfa and maintain its normal growth under water deficit conditions by improving the rhizosphere environment and significantly improving its physiological and biochemical response, and that this effect is clearly driven by the variety (genotype). We found that the drought-tolerant varieties already had high abundance of its rhizosphere drought-resistant bacteria under well-watered conditions, whereas the abundance of rhizosphere drought-resistant bacteria of drought-sensitive varieties started to increase only after being subjected to drought stress. Thus, drought-tolerant varieties can respond to drought stress in a timely and more efficient manner, whereas drought-sensitive varieties need more time to respond to drought stress and are therefore more susceptible to damage. Overall, our study provides some new insights into the importance of soil microbiomes in improving drought tolerance in alfalfa and cultivar-driven rhizosphere bacterial variation under drought stress conditions. We consider that in the future, we will try to isolate drought-tolerant rhizosphere bacteria from drought-tolerant varieties for future development as PGPR inoculants. In addition, it is important to consider the influence of microbiomes on plant yield and stress tolerance when breeding new varieties with the rhizosphere microbiome as a selective trait. Future efforts to clarify the mechanisms of recruitment of drought-tolerant bacteria in drought-tolerant varieties in terms of gene regulation and rhizosphere secretions are recommended to lay the foundation for future breeding of new varieties that can autonomously recruit beneficial bacteria against environmental stresses using biological breeding.

## Data availability statement

The datasets presented in this study can be found in online repositories. The names of the repository/repositories and accession number(s) can be found at: https://www.ncbi.nlm.nih.gov/, PRJNA900679 (SRR22266587- SRR22266604).

## Author contributions

WF designed the experiments, analyzed the data, and wrote the manuscript. WF, JW, and JX conducted the experiments. JX and JD performed the data interpretation. JD revised the manuscript critically. FT provided the experimental materials and technical support. FS conceived the experiment and manuscript revision. All authors contributed to the article and approved the final manuscript for submission.

## Funding

This work was supported by the National Natural Science Foundation of China (grant no. 32160323), Key Projects in Science and Technology of Inner Mongolia (grant no. 2021ZD0031), and the major demonstration project of “the open competition” for Seed Industry Science and Technology Innovation in Inner Mongolia (grant no. 2022JBGS0016).

## Conflict of interest

The authors declare that the research was conducted in the absence of any commercial or financial relationships that could be construed as a potential conflict of interest.

## Publisher’s note

All claims expressed in this article are solely those of the authors and do not necessarily represent those of their affiliated organizations, or those of the publisher, the editors and the reviewers. Any product that may be evaluated in this article, or claim that may be made by its manufacturer, is not guaranteed or endorsed by the publisher.

## Footnotes

^1^ http://www.biocloud.net/
