# Supplementary material for: Drought-induced recruitment of specific root-associated bacteria enhances adaptation of alfalfa to drought stress
Source: Front Microbiol. 2023 Feb 23;14:1114400. doi: 10.3389/fmicb.2023.1114400 (PMC9995459; doi:10.3389/fmicb.2023.1114400)
Supplement: Supplementary file 8 [file Image_1.PDF]

Supplementary Material

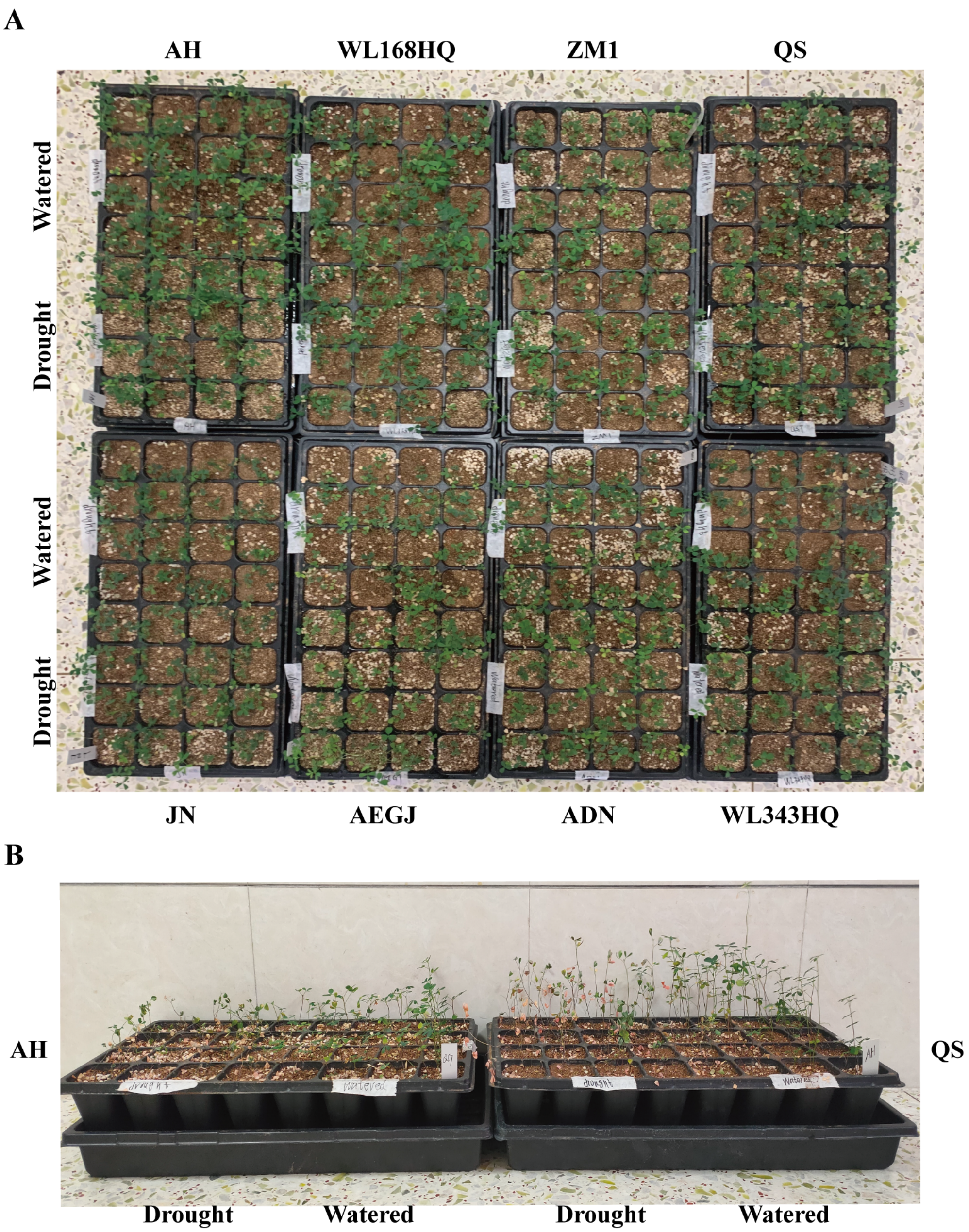

Supplementary Figure 1. Drought resistance phenotype of eight alfalfa varieties. **(A)** Growth phenotypes of eight alfalfa varieties under well-watered and drought stress for 12 days. **(B)** Growth Phenotypes of AH and QS under well-watered and drought stress for 18 days.

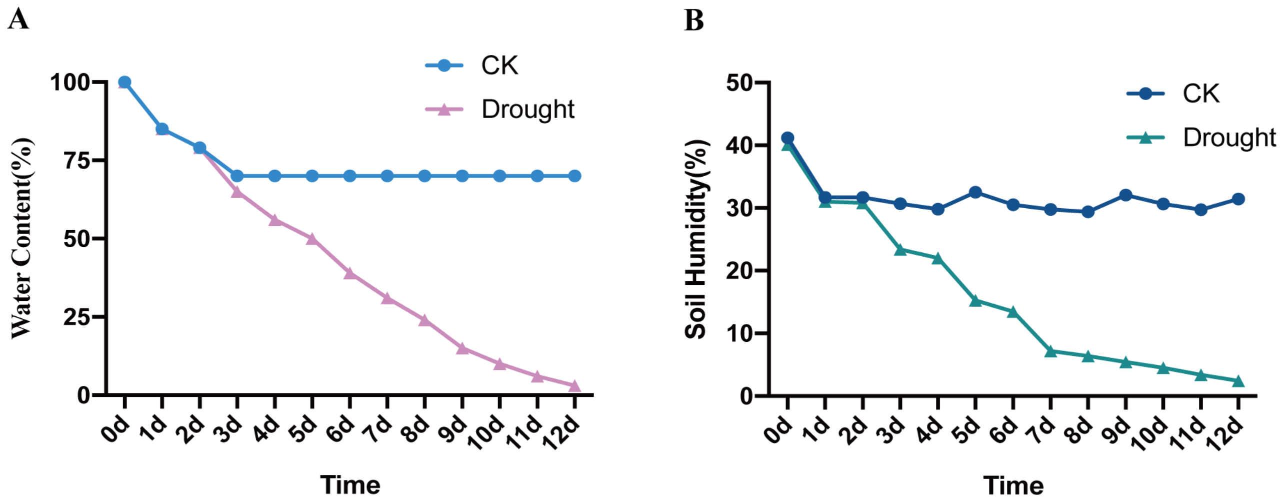

Supplementary Figure 2. Changes of soil water conditions under well-watered and drought stress for 12 days. (A) Change of water content with treatment time. (B) Change of soil humidity with treatment time.
